# The effects on depression of Internet-administered behavioural activation and physical exercise with treatment rationale and relapse prevention: study protocol for a randomised controlled trial

**DOI:** 10.1186/1745-6215-14-35

**Published:** 2013-02-02

**Authors:** Per Carlbring, Philip Lindner, Christopher Martell, Peter Hassmén, Lars Forsberg, Lars Ström, Gerhard Andersson

**Affiliations:** 1 Department of Psychology, Stockholm University, 10691, Stockholm, Sweden; 2 Department of Clinical Neuroscience, Karolinska Institutet, 17176, Stockholm, Sweden; 3 Department of Psychology, Umeå University, 901 87, Umeå, Sweden; 4 Martell Behavioral Activation Research Consulting and Department of Psychology, University of Wisconsin, Milwaukee, WI, USA; 5 Department of Behavioural Sciences and Learning, Linköping University, Linköping, Sweden

**Keywords:** Depression, Behavioural activation, Physical exercise, Treatment rationale, Relapse prevention, Internet-administered

## Abstract

**Background:**

Despite their potential as low-threshold, low-cost and high-flexibility treatments of depression, behavioural activation and physical exercise have not yet been directly compared. This study will examine the effects of these interventions, administered via the Internet. The added effect of providing a treatment rationale will also be studied, as well as a relapse prevention program featuring cognitive behavioural therapy components.

**Methods/Design:**

This randomised controlled trial will include 500 participants meeting the diagnostic criteria for major depression, recruited in multiple cycles and randomised to either a waiting list control group with delayed treatment, or one of the four treatment groups: (1) physical exercise without a clear treatment rationale; (2) physical exercise with treatment rationale; (3) behavioural activation with treatment rationale; or (4) behavioural activation without a clear treatment rationale. Post treatment, half of the participants will be offered a relapse prevention program. Primary outcome measure will be the Patient Health Questionnaire 9-item. Secondary measures include diagnostic criteria for depression, as well as self-reported anxiety, physical activity and quality of life. Measurements - done via telephone and the Internet - will be collected pre-treatment, weekly during treatment period, immediately post treatment and then monthly during a 24-month follow-up period.

**Discussion:**

The results of this study will constitute an important contribution to the body of knowledge of the respective interventions. Limitations are discussed.

**Trial registration:**

ClinicalTrials.gov: NCT01619930

## Background

It is well recognised in clinical and research settings that patients with depression account for a large portion of those seeking healthcare. In a large study of the 12-month and lifetime prevalence of mental disorders among the European population, major depression was found to be the single most common disorder with a lifetime prevalence of 12.8% and a 12-month equivalence of 3.9% [1]. In the year 2020 it is expected that depression will be the world-wide second largest disease-related cause of disability, second only to heart disease [2]. In addition to the personal suffering involved, depression is a major source of economic strain for society. The total annual cost of depression in Europe in 2004 has been estimated to €118 billion, corresponding to 1% of the total European economy at the time [3].

Treatments of depression include both pharmaceutical (antidepressants) and psychological (psychotherapy), with research showing near-equal efficacy between them [4]. Unfortunately, research also indicates that far from all who suffer from depression seek treatment [5]. A wider treatment arsenal including low-cost, high-flexibility treatments is therefore desirable. One promising treatment is activation, two kinds of which are behavioural activation, and one solely focused on physical exercise. These two kinds of activation therapy have the potential to be administered via the Internet, which would both lower the threshold for those seeking treatment, while also providing effective treatment to a low cost. In this therapeutic context, physical exercise can be considered a focused form of behavioural activation [6] in the respect that both interventions require the client to schedule and perform activities. A recent innovative randomised controlled trial comparing aerobic exercise to low-intensity stretching - both treatment arms having equal levels of activity frequency and social interaction - found no between-group difference in antidepressant effect [7]. An explanation for this result could be that it was the common activation component that caused the antidepressant effect, rather than the physical exercise *per se*. At present it is unknown whether any antidepressant effects of behavioural activation and physical exercise are treatment-specific or due to the common activation factor. Using an advanced trial design, the study herein described will be able to evaluate the antidepressant effects of both the respective treatments in themselves, and compare the two.

Strategies now known as behavioural activation (BA), originally developed by Lewinsohn and colleges in the 1970s, focused on pleasant events scheduling and BA is now defined as a structured, brief psychotherapeutic method to: (1) increase engagements in adaptive (appetitive) activities; (2) decrease engagements in activities that maintain or increase depression; and (3) increase access to reward [8]. A 2007 meta-analysis found BA to have a large effect on depressive symptoms, with the added benefit of being relatively uncomplicated, time-efficient and not requiring complex skills of patient or therapist [9]. Explaining the BA treatment rationale to patients is widely considered an early step in treatment [6], yet the component-specific effect of providing this rational has not yet been the subject of research. Acknowledging the well-known effect on outcome in cognitive behavioural therapy of the client’s acceptance of treatment rationale [10], this study will be able to distinguish the effect of providing a clear and extensive treatment rationale in BA therapy. This study will also be the first to test whether BA therapy can be adequately administered via the Internet (see [6,11]).

A 2007 Cochrane review found physical exercise to have a large clinical effect on depression. If however the methodologically weak studies were excluded, the clinical effect was moderate and non-significant [12]. Acknowledging the methodological weaknesses of previous studies, a more recent meta-analysis including only randomised controlled trials suggests a large and significant effect of physical exercise on depression [13]. It should be noted that the causality of physical exercise as treatment of depression has been questioned. In a large population-based study, De Moor *et al*. [14] found that in genetically identical twins, the twin who exercised more did not display fewer symptoms of depression, suggesting that the causal role should be ascribed to a common genetic factor rather than the exercise itself. Indeed, the specific mechanism by which physical exercise has an effect on depression, if any, is still unknown. As is the case with BA therapy, no previous research has studied the unique, component-specific effect of providing a clear treatment rationale. Providing this rationale may increase the patient’s motivation to exercise, which in itself may give a direct placebo-type antidepressant effect and/or indirectly reduced depressive symptoms following increased physical activity (which can be measured). This trial will be the first to study the component-specific effect of providing a clear treatment rationale in physical exercise therapy for depression.

In addition to studying the effects of Internet-administered physical exercise and BA - with and without providing a clear treatment rationale - and comparing them, this trial will include a depression relapse prevention program featuring cognitive behavioural therapy (CBT) components. Within 15 years, depression relapses may occur in as much as 90% of those experiencing an acute depression episode [15]. Psychotherapeutic interventions have been shown to effectively reduce the risk of depression relapses [16], yet no previous study has examined whether this type of intervention can be administered via the Internet (see [11]). With there being ample evidence for the effects of Internet-based CBT on depression [17], a CBT-style relapse prevention program delivered via the Internet holds great potential. This trial will feature such a program, provided after the physical exercise or BA intervention.

## Methods/Design

### Design

**Figure 1:**
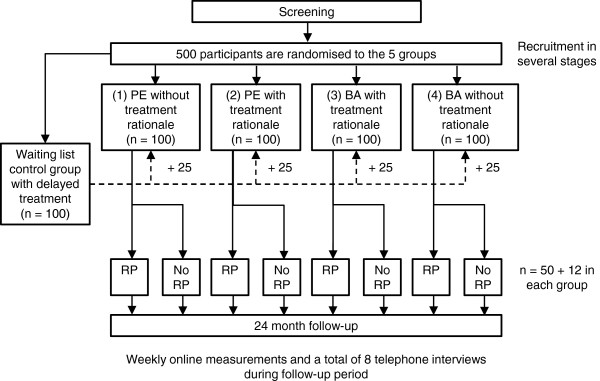
**Flow diagram of the trial. **PE, physical exercise; BA, behavioural activation; RP, relapse prevention program.

This randomised controlled trial has been registered in clinicaltrials.gov (NCT01619930). It will feature four treatment groups and a waiting list control group with delayed treatment: (1) physical exercise without treatment rationale; (2) physical exercise with treatment rationale; (3) BA with treatment rationale; (4) BA without treatment rationale; and (5) a waiting list control group, the participants of which will be randomised evenly to the four treatment groups after a 12-week delay. Participants will be recruited in several cycles over an estimated period of 1 year, with the participants of every cycle being evenly distributed to the five groups. Even distribution of participants to the different cycles will be pursued. After the treatment period of 12 weeks, 50% of each of the four treatment groups will receive a relapse prevention program. All participants will be monitored for 24 months post treatment (Figure 1).

### Screening and participants

Participants will be recruited nation-wide by means of advertisements in press and on the Internet. Application is done online, where prospective participants will also find further information on the study as well as the questionnaires serving as screening instruments. Before final inclusion, participants are required to provide written, informed consent.

The study population will consist of adults with diagnosed major depression according to DSM-IV-TR [18] criteria, with depression being the primary diagnosis. A score of 15 to 35 on the self-rated version of the Montgomery-Åsberg Depression Rating Scale (MADRS-S) [19] is also required. Other inclusion criteria include: living in Sweden and being able to read Swedish; being at least 18 years old; and having access to a computer with an Internet connection. Use of psychoactive medication is not grounds for non-inclusion if the dosage has been stable for the past 3 months and will continue to be stable during the intervention period. Any changes in medication during the study period will be noted along with the other measurements at treatment completion and at the 12- and 24-month follow-ups. If the proportion of patients who use pharmacotherapy differs between the treatment groups we will use pharmacotherapy metrics as factors in the mixed model analysis to adjust for a possible confounder effect when treatment effects are studied. Individuals meeting diagnostic criteria for treatment-demanding additional psychiatric conditions (for example, schizophrenia and bipolarity) will be excluded and referred to healthcare services. Individuals deemed suicidal or too severely depressed will also be excluded, the latter indicated by the scoring of item 9 on MADRS-S concerning the risk of suicide. When in doubt, a telephone interview will be conducted. Individuals currently receiving other psychological treatment will be excluded.

Initial selection of participants will be based on the results of the online screening instruments, consisting of MADRS-S, PHQ-9 [20], IPAQ [21], QOLI [22], GAD-7 [23] (see below) and a number of additional questions on demographics and previous treatment experiences. While the other instruments return as outcome measures (see below), MADRS-S will only be used in the screening procedure as a measure of the severity of depression symptoms. Previous research has shown that MADRS-S has a good internal consistency (Cronbach’s alpha 0.85 to 0.94), a satisfactory singe-component factor structure (47% to 69% variability explanation power), and a good correlation (Pearson’s correlation 0.81 to 0.91) with the clinician-rated version [24]. The psychometric properties of the Internet-administered version of MADRS-S has found to be sufficiently similar to the pen and paper version [25,26].

Prospective participants meeting initial inclusion criteria will be interviewed via telephone according to the Mini-International Neuropsychiatric Interview (MINI) [27] manual. Diagnostic interviews conducted per telephone have previously been found to be in very good agreement with face-to-face interviews regarding major depressive disorder [28]. The screening interviews will be conducted by supervised, clinical psychology master’s students, trained in the use of MINI. The interviewers will meet together with experienced clinical psychologists and psychiatrists to discuss final inclusion and to tailor the respective treatment program to each participant. Randomisation of participants to the different groups will be done by a statistician not otherwise involved in the study, using a computer-generated list with variables not known to the principle researchers. Participants who drop out will not be replaced.

Participants will access treatment programs, register data and communicate with their therapists using an SSL-encrypted online interface. Participants will log in using their anonymous participant number, a chosen password and unique, single-use codes sent by SMS or ordinary mail.

The study aims to detect a moderate difference in the primary outcome measure (see section 2.4.3 below) with 80% power and a 5% significance level (Bonferroni-adjusted). In accordance with this, the study will feature 500 participants, with 100 in each treatment group (125 after the control group is dissolved and divided) and a total of 250 (half of each of the four treatment groups) receiving relapse prevention.

### Interventions

All interventions will be administered and monitored via the online interface. Both the BA and physical exercise groups will receive therapist support, provided by the above-mentioned clinical psychology master’s students, supervised by an experienced clinical psychologist.

#### Physical exercise

Based on the screening data, an individualised exercise program, specifying intensity, frequency and duration of physical activity, will be provided for each participant in treatment groups 1 and 2, just prior to the treatment period commencing. A user-friendly pulse-watch, and/or pedometer will also be provided. Participants register their physical activity and receive automated, individualised feedback. The physical exercise intervention will run for 12 weeks, divided into 12 weekly modules.

#### Behavioural activation (BA)

The BA intervention provided to groups 3 and 4 will feature a variety of behaviour change strategies, including mapping of current behaviour, finding reinforcing activities and creating a difficulty-ranked hierarchy of these activities, activity scheduling and structuring, self-monitoring of mood and activity, and so on. Each participant will receive approximately 15 min of therapist support per week. The BA intervention will run for 12 weeks, divided into 12 weekly modules.

#### BA treatment rationale

The treatment rationale provided to treatment group 3 will be similar to that used in a popular self-help book [29] for treatment of depression. According to this rationale, individuals suffering from depression are trapped in a Trigger-Response-Avoidance-Pattern: a vicious circle of avoidance leading to less and less reinforcing stimuli. In accordance with this, BA serves to break the vicious circle and thereby relieve the individual of depressive suffering. Treatment group 4, which will not be provided with the TRAP rationale, will instead be informed that previous research indicates that activation improves wellbeing. Group 4 will receive the same amount of therapist support as group 3.

#### Physical exercise treatment rationale

Both groups 1 and 2 will be informed that physical exercise has been shown to decrease depression and especially fatigue, one of the core symptoms of depression. In addition to this, group 2 will also be provided with a fuller treatment rationale, according to which depression should be seen in the context of a vicious circle of lessened activity and energy levels. Both groups will receive the same amount of therapist support.

#### Control group

The waiting list control group will perform weekly measurements and receive automated, individualised feedback in graphic and message form comparing the recent results with those from previous weeks. After a 12-week delay, the group members will be randomised to one of the four treatment groups and receive treatment accordingly.

#### Relapse prevention

After the 12 treatment weeks and immediate post-treatment measurements, 50% of each treatment group of each recruitment cycle will be offered a relapse prevention program. The program will be based on and be similar to that used in a previous study [30] on relapse prevention for people suffering residual depressive symptoms after having previously received either psychotherapy or anti-depressant medication. The Internet-administered, therapist-assisted, progressive module-based program featured many of the standard components of cognitive behavioural therapy, including psychoeducation, BA, goal formation and mindfulness exercises.

### Instruments

All questionnaires will be administered via the Internet except for the clinical interviews, which will be conducted via telephone. Both these administration formats are considered valid methods of acquiring data [28,31].

#### Demographic and attitude data

At screening, data on sex, age, marital status, educational level, and so on will be collected to allow analysis of factors influencing treatment outcome and willingness to seek treatment. For the same purpose, ratings of the participants’ views on treatment credibility and expected results will also be recorded at screening using the five-item Treatment Credibility Scale (TCS) [32]. Participants will be administered the TCS for both the BA intervention and the physical exercise intervention, separately, after having read a brief description of the two interventions. Based on the parity of their participant ID number, the participants will then be randomised to either answer the TCS about BA first and then the same five questions about physical exercise, or *vice versa*. Participants will also answer what intervention they would prefer and to what degree.

#### Diagnosis

Initial diagnosis of depression, according to DSM-IV criteria, will be made using part A of the structured Mini-International Neuropsychiatric Interview (MINI) [27]. Additionally, each follow-up month, 33% of each treatment group will be interviewed per telephone. Hence, on an individual level, interviews will be conducted every third month during the follow-up period of 24 months (namely, eight follow-up interviews in total for each participant). On a group level, interviews will be conducted every month. The built-in data-loss of 66% each month will however be data missing completely at random since the participants are randomised to their respective months, making the procedure unbiased (see [33]).

#### Primary outcome

PHQ-9 [20] is the depression module of the Patient Health Questionnaire (PHQ), designed to score each of the nine DSM-IV criteria for depression as ‘0 (not at all)’ to ‘3 (nearly every day)’, allowing it to be used as both a diagnostic instrument and symptom severity scale. PHQ-9 has been shown to have good internal consistency (Cronbach’s alpha 0.86 to 0.89), good diagnostic validity [34,35] and adequate sensitivity to be used as an outcome measure [36,37]. Participants will complete the PHQ-9 weekly during the treatment period, immediately upon completion of treatment and then monthly during follow-up (24 months).

#### Secondary outcomes

In addition, three secondary outcome measures will be used. The International Physical Activity Questionnaire (IPAQ) [21] was designed to be a cross-nationally valid self-reported measure of physical activity, and features questions on the amount of time (minutes, hours and days) spent in the last 7 days on physical activity and sitting still. IPAQ will be administered upon treatment completion and thereafter at the 12- and 24-month follow-ups.

The seven-item general anxiety scale, GAD-7 is used for assessing anxiety and screening for generalised anxiety disorder, with psychometric evaluation having yielded good internal consistency (Cronbach’s alpha 0.92) and a good factorial structure (69% to 81% of variance explained) [23]. GAD-7 will be administered upon treatment completion and thereafter monthly during the follow-up period.

The Quality Of Life Inventory (QOLI) [22] will also be included. QOLI features 32 items ranging over 16 areas of life (health, economy, work, and so on) and has good internal consistency (Cronbach’s alpha 0.77 to 0.89) and test-retest reliability (0.80 to 0.91) [22]. QOLI will be administered upon treatment completion and thereafter at the 12- and 24-month follow-ups.

At screening, as well as immediate after treatment and at the 12- and 24-month follow-ups, participants will also answer the EQ-5D [38] to assess generic health status, and the Trimbos/iMTA questionnaire for Costs associated with Psychiatric illness (TiC-P) [39] to assess health economic aspects.

### Analysis

The study design will allow for multiple comparisons: the two physical exercise groups (separately and together) will be compared to controls, as will the two BA groups (separately and together). In all other analyses, the groups in question will include those from the control group later randomised to interventions, this to increase the statistical power. The two physical exercise groups will be contrasted with the two BA groups. Further, the physical exercise group with treatment rationale (group 1) will be contrasted with the group without (group 2), and the BA group with treatment rationale (group 3) will be contrasted with the BA group without (group 4). Finally, the four groups receiving relapse prevention will be contrasted with the four groups that do not. At the 12- and 24-month follow-ups, within-group comparisons will be made with previous results.

A number of statistical analyses will be deployed. Treatment outcomes will be examined using mixed effect models, Bonferroni-correcting for multiple comparisons. This method is deemed preferable to uni- and multivariate repeated measures of variance [40], especially since the study has built-in data-loss. The Kaplan-Meier estimator will be used to estimate survival rates during the study period. To find potential predictors of treatment outcomes (including treatment adherence and drop-out), logistic regression analysis on background variables will be performed (see [41]).

Since all questionnaire data will be supplied directly by the participants through the online interface, there is no risk of data loss or data distortion along the way. Data will be stored encrypted and in unidentifiable form (using participant-numbers). Standard missing data analysis will determine if unexpected missing data due to participant drop-out are random or not.

### Ethics

This study has been approved by the regional Ethical Board. As in any trial, there are a number of ethical concerns, all of which will be adequately addressed. First and foremost, great care will be taken not to include participants too severely depressed or suffering from other disorders better treated elsewhere. Should suspicion arise during the intervention period that an enrolled participant is experiencing a significant decline in health due to the intervention, the supervising clinicians (licensed psychologists, psychotherapists or psychiatrist) may opt to end the participation prematurely and direct the participant to other healthcare services. No adverse side effects of BA or physical exercise are expected, yet side effects will nonetheless be investigated by asking standard treatment side effects questions at the post-treatment measurements, in accordance with the recommendations by Linden [42]. As in all internet interventions, the privacy of the participants is of paramount importance [43]. In this trial, participants will use anonymous participant codes to interact with a secure online interface after a two-stage log-in procedure.

## Discussion

This protocol describes a large (*n* = 500) randomised controlled trial examining the effects on depression of internet-administered behavioural activation and physical exercise programs, with relapse prevention and treatment rationale as additional distinguishable components. As the economic strain and personal suffering associated with depression continues to increase, so does the need for a variety of treatment methods. Despite their potentials as low-threshold, low-cost, high-flexibility treatments of depression, behavioural activation and physical exercise have yet to be the subject of much research. Any future integration of behavioural activation and physical exercise into healthcare systems will of course require scientific evidence of the effects of these treatments. The study herein described will make substantial contributions to the body of knowledge on these areas, while also examining the separate effects of providing a clear treatment rationale. The potential of a CBT-style program to prevent depression relapse will also be examined. The randomised controlled design is stringent throughout the study, allowing valid conclusions on the effects of the respective interventions. To our best knowledge, this study is also the first to feature Internet-administered behavioural activation; the results will consequently provide a first indication whether the treatment can be adequately translated to this promising administration format.

This study has some limitations that need to be recognised. For ethical reasons, individuals suffering from too severe depression, with or without the risk of suicide, will not be included. Since the participants in the waiting list control group will receive treatment after a 12-week delay, this study will lack a control group in the follow-up period. This again is for ethical reasons. As in almost all studies featuring psychotherapeutic interventions, blinding participants and researchers is not feasible. A final aspect in need of recognition is the involvement of the master’s students in clinical psychology. To compensate for the students’ lack of training and experience, an experienced licensed clinical psychologist and psychotherapist will supervise all involvement.

## Trial status

Recruitment is scheduled to begin in January 2013. The participants of the first recruitment cycle are planned to commence treatment later that spring.

## Abbreviations

BA: Behavioural activation; CBT: Cognitive behavioural therapy; DSM-IV-TR: Diagnostic and statistical manual of mental disorders, fourth text-revised edition; GAD-7: Generalized Anxiety Disorder 7; IPAQ: International Physical Activity Questionnaire; MADRS-S: Montgomery-Åsberg Depression Rating Scale Self-rated; MINI: Mini-International Neuropsychiatric Interview; PHQ-9: Patient Health Questionnaire 9; RP: Relapse prevention program; QOLI: Quality Of Life Inventory; TCS: Treatment Credibility Scale; TICP-P: Trimbos/iMTA questionnaire for Costs associated with Psychiatric illness.

## Competing interests

Six out of the seven authors declare that they have no competing interests. CM has written a self-help book similar to the treatment that will be in one of the treatment arms (BA). Consequently, CM will not be involved in any of the informed consent procedures or analyses of outcome data.

## Authors’ contributions

PC in collaboration with GA, CM, PH and LF designed the study. PL and LS made critical contributions to the conception of the study. PL drafted the manuscript. All authors participated in the review and revision of the manuscript and have approved the final manuscript to be published.

## References

[B1] Alonso J Angermeyer MC Bernert S Bruffaerts R Brugha TS Bryson H De Girolamo G Graaf R Demyttenaere K Gasquet I Haro JM Katz SJ Kessler RC Kovess V Lépine JP Ormel J Polidori G Russo LJ Vilagut G Almansa J Arbabzadeh-Bouchez S Autonell J Bernal M Buist-Bouwman M Codony M Domingo-Salvany A Ferrer M Joo SS Martínez-Alonso M Matschinger H Prevalence of mental disorders in Europe: results from the European Study of the Epidemiology of Mental Disorders (ESEMeD) project Acta Psychiatr Scand Suppl 2004 109 21 27 15128384 10.1111/j.1600-0047.2004.00327.x

[B2] Murray CJL Lopez AD Evidence-based health policy-lessons from the Global Burden of Disease Study Science 1996 274 740 743 10.1126/science.274.5288.740 8966556

[B3] Sobocki P Jonsson B Angst J Rehnberg C Cost of depression in Europe J Ment Health Policy Econ 2006 9 87 98 17007486

[B4] Spielmans GI Berman MI Usitalo AN Psychotherapy versus second-generation antidepressants in the treatment of depression: a meta-analysis J Nerv Ment Dis 2011 199 142 149 10.1097/NMD.0b013e31820caefb 21346483

[B5] Mojtabai R Unmet need for treatment of major depression in the United States Psychiatric services (Washington, D.C.) 2009 60 297 305 10.1176/appi.ps.60.3.297 19252041

[B6] Veale D Behavioural activation for depression Adv Psychiatr Treat 2008 14 29 36 10.1192/apt.bp.107.004051

[B7] Krogh J Videbech P Thomsen C Gluud C Nordentoft M DEMO-II trial. Aerobic exercise versus stretching exercise in patients with major depression-a randomised clinical trial PLoS One 2012 7 e48316 10.1371/journal.pone.0048316 23118981 PMC3485141

[B8] Dimidjian S Barrera M Martell C Muñoz RF Lewinsohn PM The origins and current status of behavioral activation treatments for depression Annu Rev Clin Psychol 2011 7 1 38 10.1146/annurev-clinpsy-032210-104535 21275642

[B9] Cuijpers P Van Straten A Warmerdam L Behavioral activation treatments of depression: a meta-analysis Clin Psychol Rev 2007 27 318 326 10.1016/j.cpr.2006.11.001 17184887

[B10] Addis M Jacobson N A closer look at the treatment rationale and homework compliance in cognitive-behavioral therapy for depression Cogn Ther Res 2000 24 313 326 10.1023/A:1005563304265

[B11] Andersson G Carlbring P Berger T Almlov J Cuijpers P What makes Internet therapy work? Cogn Behav Ther 2009 38 55 60 10.1080/16506070902916400 19675956

[B12] Mead GE Morley W Campbell P Greig CA Marion McMurdo DAL Exercise for depression 2009 Chichester: John Wiley & Sons, Ltd

[B13] Rethorst CD Wipfli BM Landers DM The antidepressive effects of exercise: a meta-analysis of randomized trials Sports Med 2009 39 491 511 10.2165/00007256-200939060-00004 19453207

[B14] De Moor MHM Boomsma DI Stubbe JH Willemsen G De Geus EJC Testing causality in the association between regular exercise and symptoms of anxiety and depression Arch Gen Psychiatry 2008 65 897 905 10.1001/archpsyc.65.8.897 18678794

[B15] Keller MB Long-term treatment of recurrent and chronic depression J Clin Psychiatry 2001 Suppl 2 3 5 11676430

[B16] Nierenberg A Petersen TJ Alpert JE Prevention of relapse and recurrence in depression: the role of long-term pharmacotherapy and psychotherapy J Clin Psychiatry 2003 Suppl 1 13 17 14658986

[B17] Johansson R Andersson G Internet-based psychological treatments for depression Expert Rev Neurother 2012 12 861 869 10.1586/ern.12.63 22853793

[B18] America Psychiatric Association Diagnostic and statistical manual of mental disorders: DSM-IV-TR 2000 4 Arlington, VA: APA

[B19] Svanborg P Åsberg M A new self-rating scale for depression and anxiety states based on the Comprehensive Psychopathological Rating Scale Acta Psychiatr Scand 1994 89 21 28 8140903 10.1111/j.1600-0447.1994.tb01480.x

[B20] Kroenke K Spitzer RL Williams JBW The PHQ-9: validity of a brief depression severity measure J Gen Intern Med 2001 16 606 613 10.1046/j.1525-1497.2001.016009606.x 11556941 PMC1495268

[B21] Craig CL Marshall AL Sjöström M Bauman AE Booth ML Ainsworth BE Pratt M Ekelund U Yngve A Sallis JF Oja P International physical activity questionnaire: 12-country reliability and validity Med Sci Sports Exerc 2003 35 1381 1395 10.1249/01.MSS.0000078924.61453.FB 12900694

[B22] Frisch MB Cornell J Villanueva M Retzlaff PJ Clinical validation of the Quality of Life Inventory. A measure of life satisfaction for use in treatment planning and outcome assessment Psychol Assess 1992 4 92 101

[B23] Spitzer RL Kroenke K Williams JBW Löwe B A brief measure for assessing generalized anxiety disorder: the GAD-7 Arch Intern Med 2006 166 1092 1097 10.1001/archinte.166.10.1092 16717171

[B24] Bondolfi G Jermann F Rouget BW Gex-Fabry M McQuillan A Dupont-Willemin A Aubry J-M Nguyen C Self- and clinician-rated Montgomery-Asberg Depression Rating Scale: evaluation in clinical practice J Affect Disord 2010 121 268 272 10.1016/j.jad.2009.06.037 19660815

[B25] Hedman E Ljótsson B Rück C Furmark T Carlbring P Lindefors N Andersson G Internet administration of self-report measures commonly used in research on social anxiety disorder: A psychometric evaluation Comput Hum Behav 2010 26 736 740 10.1016/j.chb.2010.01.010

[B26] Holländare F Andersson G Engström I A comparison of psychometric properties between internet and paper versions of two depression instruments (BDI-II and MADRS-S) administered to clinic patients J Med Internet Res 2010 12 e49 10.2196/jmir.1392 21169165 PMC3057311

[B27] Sheehan D Lecrubier Y Sheehan K Amorim P Janavs J Weiller E Hergueta T Baker R Dunbar G The Mini-International Neuropsychiatric Interview (M.I.N.I.): the development and validation of a structured diagnostic psychiatric interview for DSM-IV and ICD-10 J Clin Psychiatry 1998 Suppl 20 22 33 9881538

[B28] Rohde P Ph D Lewinsohn PM Seeley JR Comparability of telephone and face-to-face interviews in assessing axis I and II disorders Am J Psychiatry 1997 154 1593 1598 9356570 10.1176/ajp.154.11.1593

[B29] Addis M Martell C Overcoming Depression One Step at a Time 2004 Oakland, CA: New Harbinger Publications 190

[B30] Holländare F Johnsson S Randestad M Tillfors M Carlbring P Andersson G Engström I Randomized trial of Internet-based relapse prevention for partially remitted depression Acta Psychiatr Scand 2011 124 285 294 10.1111/j.1600-0447.2011.01698.x 21401534

[B31] Thorndike FP Carlbring P Smyth FL Magee JC Gonder-Frederick L Ost L-G Ritterband LM Web-based measurement: Effect of completing single or multiple items per webpage Comput Hum Behav 2009 25 393 401 10.1016/j.chb.2008.05.006

[B32] Borkovec TD Nau SD Credibility of analogue therapy rationales J Behav Ther Exp Psychiatry 1972 3 257 260 10.1016/0005-7916(72)90045-6

[B33] Yoon FB Fitzmaurice GM Lipsitz SR Horton NJ Laird NM Normand S-LT Alternative methods for testing treatment effects on the basis of multiple outcomes: simulation and case study Stat Med 2011 30 1917 1932 10.1002/sim.4262 21538986 PMC3116112

[B34] Löwe B Gräfe K Zipfel S Witte S Loerch B Herzog W Diagnosing ICD-10 depressive episodes: superior criterion validity of the Patient Health Questionnaire Psychother Psychosom 2004 73 386 390 10.1159/000080393 15479995

[B35] Löwe B Spitzer RL Gräfe K Kroenke K Quenter A Zipfel S Buchholz C Witte S Herzog W Comparative validity of three screening questionnaires for DSM-IV depressive disorders and physicians’ diagnoses J Affect Disord 2004 78 131 140 10.1016/S0165-0327(02)00237-9 14706723

[B36] Löwe B Unützer J Callahan CM Perkins AJ Lwe B Uniitzer J Kroenke K Monitoring depression treatment outcomes with the patient health questionnaire-9 Medical Care 2004 42 1194 1201 10.1097/00005650-200412000-00006 15550799

[B37] Löwe B Kroenke K Herzog W Gräfe K Measuring depression outcome with a brief self-report instrument: sensitivity to change of the Patient Health Questionnaire (PHQ-9) J Affect Disord 2004 81 61 66 10.1016/S0165-0327(03)00198-8 15183601

[B38] Brooks R Rabin R De Charro F The Measurement and Valuation of Health Status Using EQ-5D: A European Perspective: Evidence from the EuroQol BIO MED Research Programme 2003 Rotterdam: Kluwer Academic Publishers

[B39] Haakkart-van Roijen L Van Straten A Donker M Tiemens B Manual Trimbos/iMTA questionnaire for Costs associated with Psychiatric illnes (TiC-P) 2002 Rotterdam: Institute for Medische Technology Assessment, Erasmus University Rotterdam

[B40] Gueorguieva R Krystal JH Move over ANOVA: progress in analyzing repeated-measures data and its reflection in papers published in the Archives of General Psychiatry Arch Gen Psychiatry 2004 61 310 317 10.1001/archpsyc.61.3.310 14993119

[B41] Andersson G Bergström J Holländare F Ekselius L Carlbring P Delivering cognitive behavioural therapy for mild to moderate depression via the Internet: predicting outcome at 6-month follow-up Verhaltenstherapie 2004 14 185 189 10.1159/000080914

[B42] Linden M How to define, find and classify side effects in psychotherapy: from unwanted events to adverse treatment reactions Clin Psychol Psychother 2012 Jan 18 Epub ahead of print 10.1002/cpp.1765 22253218

[B43] Proudfoot J Klein B Barak A Carlbring P Cuijpers P Lange A Ritterband L Andersson G Establishing guidelines for executing and reporting Internet intervention research Cogn Behav Ther 2011 40 82 97 10.1080/16506073.2011.573807 25155812

